# Novel Mutations in *AKT1* Gene in Prostate Cancer Patients in Jordan

**DOI:** 10.3390/cimb46090586

**Published:** 2024-09-04

**Authors:** Ala’a Alasmar, Zina Al-Alami, Sima Zein, Asmaa Al-Smadi, Samir Al Bashir, Mohammed S. Alorjani, Raed M. Al-Zoubi, Mazhar Al Zoubi

**Affiliations:** 1 Department of Medical Laboratory Sciences, Faculty of Allied Medical Sciences, Al-Ahliyya Amman University, Amman 19328, Jordan; alaaalasmarr97@gmail.com; 2 Department of Basic Laboratory Sciences, Faculty of Allied Medical Sciences, Al-Ahliyya Amman University, Amman 19328, Jordan; z.alalami@ammanu.edu.jo; 3 Department of Pharmaceutical Biotechnology, Faculty of Allied Medical Sciences, Al-Ahliyya Amman University, Amman 19328, Jordan; s.zein@ammanu.edu.jo; 4 Department of Basic Medical Sciences, Faculty of Medicine, Yarmouk University, Irbid 21163, Jordan; asmaasmadi@gmail.com; 5 Department of Pathology and Microbiology, Faculty of Medicine, Jordan University of Science and Technology, Irbid 22110, Jordan; smalbashir9@just.edu.jo (S.A.B.); msalorjani@just.edu.jo (M.S.A.); 6 Surgical Research Section, Department of Surgery, Hamad Medical Corporation, Doha P.O. Box 3050, Qatar; ralzoubi@hamad.qa; 7 Department of Biomedical Sciences, QU-Health, College of Health Sciences, Qatar University, Doha 2713, Qatar

**Keywords:** *AKT1*, E17K, PH domain, prostate cancer, mutations, sustainable healthcare

## Abstract

The *AKT1* oncogene is related to various cancers due to its critical role in the PIC3CA/AKT1 pathway; however, most of the studies screened the hotspot mutation *AKT1* (E17K) with various incidences. Low frequency or lack of *AKT1* (E17K) mutation was reported in prostate cancer (PC) patients. This study aims to explore genetic alterations in the AKT1 PH domain by extending the sequencing to include *AKT1* gene exons 3 and 4. Genomic DNA was extracted from 84 Formalin-Fixed Paraffin-Embedded samples of PC patients in Jordan, and then subjected to PCR and sequencing for the targeted exons. This study revealed the presence of two novel mutations (N53Y and Q59K) and a high frequency of mutations in exon 4, with a lack of mutations in the E17K hotspot. Nine missense and two synonymous mutations were detected in exon 4 (Phe27Tyr, Phe27Leu, Ala58Thr, Ser56Phe, Arg41Trp, Phe35Leu, Asp32Glu, Phe35Tyr, and Gln43Lys) and (Ser56 and Glu40), respectively. Two synonymous mutations were detected in exon 3 (Leu12 and Ser2). It is concluded that there is a high frequency of *AKT1* mutation in PC patients in Jordan with two novel missense mutations in the Pleckstrin homology (PH) domain. E17K hotspot mutation was not detected in any tested samples, which underlined the significant role of mutations in other *AKT1* exons in PC development.

## 1. Introduction

Prostate cancer (PC) is one of the most common malignancies among men, representing the first leading cause of cancer-related death in men all over the world [1], and the second most prevalent cancer in men globally [2]. PC varies among different nations and ethnic groups, with the most occurrence in North America and other Western countries. Similarly, the incidence of PC in the Middle East has increased over the last decade [3,4]. However, in the Middle East and North Africa, it appears to be less common, based on age and ethnicity. Nevertheless, a high proportion of men from the Middle East develop de novo metastasis [5]. In Jordan, the incidence of PC represents 8.5% of all new cancer cases. For instance, 451 PC cases were reported in 2020 in Jordan with 63 related deaths [6].

Only 5–10% of PC cases have been related to familial inheritance or family history [7]. Most PC is believed to be associated with many factors, such as genetic alterations, endogenous hormone balance, environmental factors, and certain health habits, particularly fatty diets [8]. Many genetic alterations in certain genes have been related to the occurrence of PC. For instance, SPOP, PTEN, PIK3CA, and RAD51 gene alterations have been reported in PC [9]. Nevertheless, many other genetic alterations and variants are proposed to be related to the occurrence of PC. *AKT1* genetic alterations were reported in some cancers, including breast cancer [10,11,12,13]. The *AKT1* gene is coded for protein kinase B, a protein that serves many functions, including the building up of proteins that are important in the cell signaling system involved in cell survival, drug resistance, cell motility and invasion, and metabolism [14,15]. The phosphoinositide 3-kinase-AKT (PI3k-AKT) pathway is one of the most commonly altered pathways in all cancers; the homeostatic balance of cell division and programmed cell death is generally disturbed in tumorigenesis, and downstream effectors of the PI3k-AKT pathway play an essential role in this process [16]. Due to its crucial functions, especially in tumor metastasis, AKT inhibition may result in affecting an important pathway to cell survival and leading to stimulation of downstream proteins, such as receptor tyrosine kinases, which permit cancer cells to survive [17].

Akt1 consists of a serine/threonine-specific kinase domain, a C-terminal regulatory domain [18], a large central kinase domain, an N-terminal Pleckstrin homology (PH) domain, and a C-terminus regulatory domain. AKT isoforms Akt1(PKBα), Akt2 (PKBβ), and Akt3 (PKBγ) share substantial similarities but are expressed from three different genes. Their expression is tissue-specific, and each has different functions; Akt1 is required for cell survival; Akt2 is more related to glucose homeostasis; and Akt3 is important in brain development [19]. Generally, AKT isoforms are enzymes that transfer the gamma-phosphoryl group from ATP to Ser473, Thr308, or Tyr [20] of target proteins [21]. The phosphorylated proteins are essential components of many signaling pathways that are involved in the regulation of cell proliferation and apoptosis, and it is critical in the initiation and progression of tumors, enhancing cell survival by stimulating cell proliferation and inhibiting apoptosis [19]

*AKT1* gene produces Akt1 kinase, which is involved in various signaling pathways required for proper development and nervous system function; it contributes to the regulation of cell growth and division, controls differentiation and cell survival, and aids in the regulation of apoptosis. Akt1 kinase has been implicated in cell-to-cell communication among neurons, neuronal survival, and development of memories [22]. *AKT1* (E17K) is a frequent somatic mutation found in several cancer types that mainly activates the PI3K/AKT signal pathway [23]. Some studies have demonstrated that this mutation can cause protein activation, which activates the PI3K/AKT/mTOR pathway in breast cancer cells [24]. The point mutation (G => A) at nucleotide 49 leads to the substitution of lysine for glutamic acid at codon 17 (E17K). The amino acid alteration changes the structure of the PH domain, causing the protein to shift from the cytoplasm to the plasma membrane. This activates the Akt1 kinase, which, in turn, activates the mTOR and ERK1/2 signaling pathways [24]. *AKT1* somatic mutations were reported in breast, ovarian [25], and colorectal [26] malignancies [27].

This study aims to evaluate the association between *AKT1* genetic variants in PC cases of Jordanian men. In particular, the study intended to sequence exons 3 and 4 of the *AKT1* gene in the selected cohort.

## 2. Materials and Methods

### 2.1. Sample Collection

This study included 100 Formalin-Fixed Paraffin-Embedded (FFPE) tissue samples from patients who underwent prostatectomy at King Abdallah University Hospital (KAUH) by anatomical pathologists from (2003–2009). Genomic DNA was successfully extracted from 84 samples. The mean age of the study cohort was 72 years (range 55–95 years). The samples in the current study were collected after being approved by the institutional review approval (IRB) from King Abdullah University Hospital (KAUH) (Irbid, Jordan), IRB# (68/124/2019).

### 2.2. DNA Extraction

Genomic DNA was extracted using ZYMO Research Quick-DNA Formalin-Fixed Paraffin-Embedded (FFPE) Miniprep kit (Irvine, CA, USA) according to the manufacturer’s instructions. Briefly, four to ten sections of FFPE tissue were deparaffinized 3 times with 1mL of Xylene and incubated at 55 °C for 5 min, followed by washing 3 times with absolute ethanol. Then, they were incubated for 2–3 h at 55 °C or overnight at room temperature to dry completely. The dry tissue was digested by 50 µL of proteinase K buffer and 400 µL digestion buffer mixed very well and incubated overnight with shaking at 55 °C, followed by incubation at 90 °C for 30–60 min. Afterward, DNA purification was performed as requested by the company’s protocol. All collected samples were stored at –20 °C for short-term use, and DNA samples were stored at −80 °C for long-term storage for further analysis. Using a NanoDrop spectrophotometer (Thermo Fisher Scientific Inc, Pittsburgh, PA, USA), the purity and concentration of the DNA were estimated, and the results were verified by BIOER XP thermal cycler model TC-E-96G (Bioer, Binjiang, China) for gene amplification.

### 2.3. Primer Design and PCR Amplification

The PCR amplification of exons 3 and 4 of *AKT1* gene were achieved by using specific sets of primers that were designed by primer3 and Blast primer websites (Chromosome 14 NC_000014.9) according to National Center for Biotechnology Information (https://www.ncbi.nlm.nih.gov/ accessed on 1 April 2024) and Ensemble genome browser (https://asia.ensembl.org/index.html accessed on 1 April 2024), as shown in Table 1. The 30 μL reaction volume for PCR amplification consisted of 3 μL of genomic DNA, 0.7 μL of (10 μM) forward primer, 0.7 μL of (10 μM) reverse primer, 19.6 μL nuclease-free water, and 6 μL of 5X master mix, which contained 12.5 MgCl_2_ from HOT FIREPol^®^ Blend Master Mix, Tartu, Estonia. The PCR amplification process was performed using the BIOER XP thermal cycler model TC-E-96G (Bioer, Binjiang, China) under the cycling conditions mentioned in Table 1.

### 2.4. DNA Sequencing

PCR products were purified and analyzed using Sanger sequencing at a local company (Biotrust laboratories, Irbid, Jordan) on a genetic analyzer (SeqStudio™ Genetic Analyzer System with SmartStart Applied Biosystems™, Thermo Fisher Scientific Inc., Pittsburgh, PA, USA). The output of sequencing was analyzed using the Mutation Surveyor (v5.2.0), Bioedit (7.7), and Unipro UGENE (50.0) software.

### 2.5. Data Analysis

The clinical-pathological data were analyzed using a *t*-test and Chi-square test, which were calculated by GraphPad Prism 9 software.

## 3. Results

Eighty-four FFPE PC samples were screened for the mutations in exons 3 and 4 of the *AKT1* gene. Generally, the results revealed that 13 samples were altered at both exons, exon 3 and exon 4 of the *AKT1* gene (15.4%). Two samples showed mutations at exon 3 (2.3%), and eleven samples showed mutations at exon 4 (13.1%). Two samples have shown double missense mutations in exon 4 (rs2140949629 and rs749781543) and (rs201636005 and rs2140949360). The detected mutations in exon 3 and exon 4 of the *AKT1* gene are summarized in Table 2 and Table 3.

**Figure 1:**
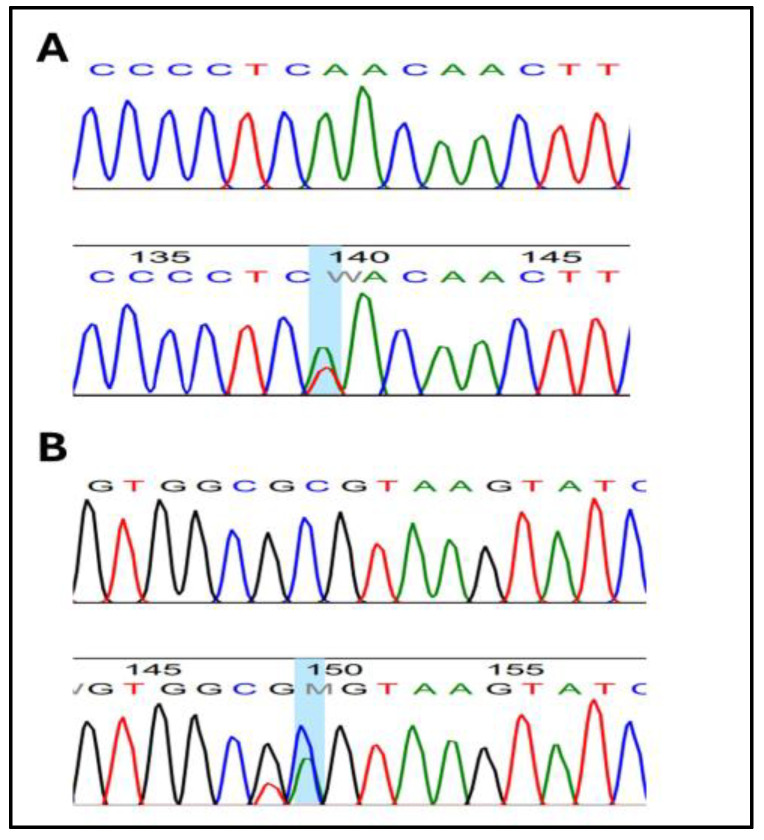
Sequence chromatograms of novel mutations recorded in exon 4; (**A**): c.157A > T and (**B**): c.175C > A (175C > A alteration is the last nucleotide in exon 4). c.174G > T is a synonymous alteration. (The figure was created using FinchTV Version 1.5.0, developed by Geospiza Inc., Denver, CO, USA). Genetic alterations are highlighted in blue.

**Figure 2:**
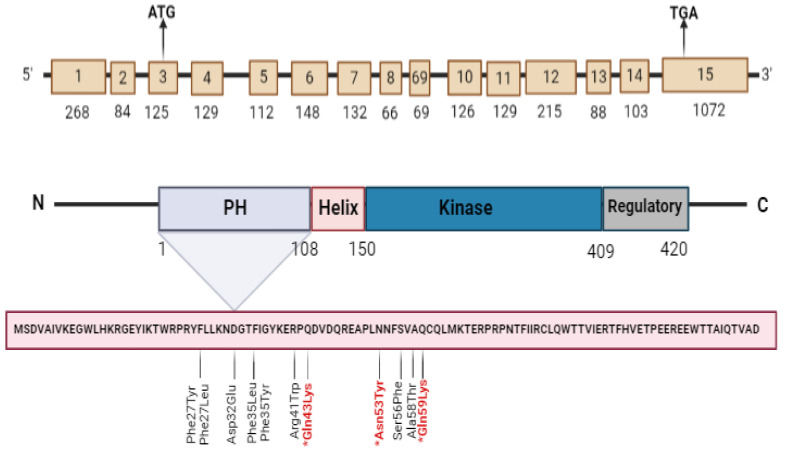
*AKT1* gene exons and introns showing relative Akt1 protein domains, amino acid sequence of PH domain, and all variants reported in exon 4. * represents the missense variants.

**Figure 3:**
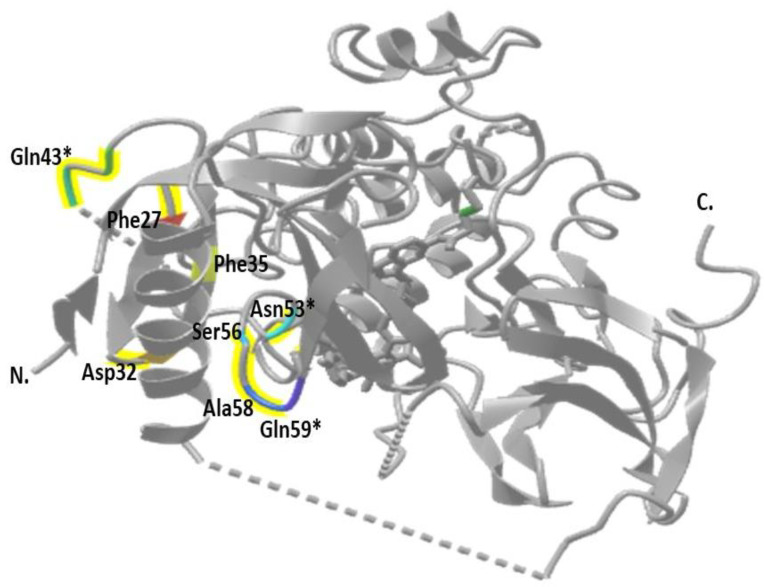
A 3D structure of Akt1 protein (RAC-alpha serine/threonine-protein kinase isoform X1) labeled with the positions of amino acid changes of all variants recorded in exon 4. * represents the missense variants.

To our knowledge and search in genetic databases, two novel variants were found in exon 4 that were not previously recorded, namely N53Y and Q59K. Using the PolyPhen2 website (http://genetics.bwh.harvard.edu/pph2/ accessed on 1 April 2024), both variants c.127C > T (Q43K) and C: c.175C > A (Q59K) mutations were predicted to be benign. In contrast, the variant c.157A > T (N53Y) appeared to be (probably ruinous) (Table 3). The sequence chromatograms of the two novel mutations are shown in Figure 1. *AKT1* gene exons and introns, showing Akt1 protein domains, amino acid sequence of PH domain, and all variants reported in exon 4, are shown in Figure 2, and a 3D structure of Akt1 protein (RAC-alpha serine/threonine-protein kinase isoform X1) labeled with the positions of amino acid changes of all variants recorded in exon 4 is shown in Figure 3.

Nine missense variants were found in exon 4, namely rs2140949629 (Phe27Tyr), rs749781543 (Phe27Leu), rs1892948718 (Ala58Thr), rs2140948579 (Ser56Phe), rs753765116 (Arg41Trp), rs2140949369 (Phe35Leu), rs201636005 (Asp32Glu), rs2140949360 (Phe35Tyr), and COSV62573749 (Gln43Lys), and two synonymous variants rs2140948571 (Ser56) and rs370287382 (Glu40).

In both exons, the correlation between PSA level with age and Gleason score was not significantly different (*p* > 0.05) (Table 4 and Table 5).

## 4. Discussion

PC is associated with many risk factors, such as environmental, ethnicity (race), and age factors. In addition, Genome-Wide Association Studies (GWASs) revealed dozens of single nucleotide polymorphisms (SNPs) correlated with the risk of PC [28]. For instance, genetic alteration in certain genes, such as BRCA2, BRCA1, HOXB13, NBS1, CHEK2, SPOP, PTEN, TP53, and many other SNPs, have been related to the risk of PC [29,30,31,32,33,34]. Nonetheless, the molecular portrait of PC is not yet revealed, giving more importance to early diagnostics and therapeutics [35]. Some studies found that individual SNPs have a low to moderate effect on PC progression compared to certain haplotypes. A better understanding of the role of SNPs may play a role in PC susceptibility, improving fine-mapping and gaining new insights into the pathophysiology of PC. Therefore, exploring certain oncogenes might reveal a stronger correlation with PC development, which will positively impact the prediction and precision medicine approach. The current study proposed *AKT1* as an important oncogene that might be associated with the risk of PC in Jordanian men.

*AKT1* is a gene found at chromosome 14q32.33; it is one of the three serine/threonine-protein kinases (AKT1, AKT2, and AKT3). In response to the stimulation of some growth factors, these kinases regulate processes such as cell survival, metabolism, proliferation, angiogenesis, and growth, as they phosphorylate a group of downstream substrates [36]. *AKT1* genetic alterations have been reported in certain cancers; however, they are limited to the detection of E17K mutation in exon 3. In the current study, exon 3 and exon 4 of the *AKT1* gene were sequenced to explore the extended rear of the PH domain. The results showed that the percentage of mutations was 15.4% in exon 3 and exon 4 without reporting any E17K point mutation, where most of the detected alterations were found in exon 4. Despite the high frequency of genetic alterations in exon 4, two novel variants were detected: N53Y and Q59K. While nine missense variants, namely rs2140949629 (Phe27Tyr), rs749781543 (Phe27Leu), rs1892948718 (Ala58Thr), rs2140948579 (Ser56Phe), rs753765116 (Arg41Trp), rs2140949369 (Phe35Leu), rs201636005 (Asp32Glu), rs2140949360 (Phe35Tyr), and COSV62573749 (Gln43Lys), and two synonymous variants rs2140948571 and rs370287382 are detected in exon 4 of the *AKT1* gene. The results emphasized the importance of extending the exploration of genetic alteration in the *AKT1* gene through the other exons rather than focusing on the common hotspot mutation E17K.

Most studies explored the incidence of the *AKT1* (E17K) hotspot mutation in many cancers where glutamic acid is replaced with lysine. The E17 point mutation is in the PH domain at the amino terminus of *AKT* genes. PH domain is critical in cell signaling, phosphoinositide binding, and protein binding [37]. The association of the E17K mutation with the studied cancer is attributed to the role of the phosphatidylinositol 3-kinase/protein kinase B (PI3K-/PKB) signaling pathway activation in certain cancers [38]. In particular, the E17K mutation causes conformational changes in the PH domain of the AKT1, changing its localization from the cytoplasm to the plasma membrane, leading to constitutive activation of AKT kinase, and consequently, the activation of mTOR and ERK1/2 signaling pathways [24].

*AKT1* (E17K) mutation was reported in breast cancer, meningioma, colorectal cancer, lung cancer, bladder, and ovarian cancer in different frequencies [10,11,12,13,24,38,39,40,41,42,43,44,45,46,47,48]. Up to 20–62% frequency of *AKT1* (E17K) mutations has been detected in the benign papillary tumor of mammary glands and is mainly found in estrogen receptor-positive breast cancer patients [10,11,12,13]. In addition, the E17K mutation was detected in two types of breast tumors: the lobular and ductal tissue type [13,40]. Moreover, in a typical early-stage ductal hyperplasia, E17K occurs more frequently than aggressive carcinoma [40]. Most non-NF2 gene meningiomas are usually caused by mutations in SMO (9%) and *AKT1* (30%), especially in the cases where the meningioma is at the convexity at the skull-based, respectively [46,47,48,49]. The frequency of *AKT1* (E17K) mutation in lung cancer is rare; it is approximately 0.6–5.5% in lung cancer patients. The E17K mutation was reported to be mainly expressed in the lung epithelium, and this mutation can only be present in squamous cell carcinoma in non-small cell lung cancer [40,43,44,50,51,52,53]. In addition, the *AKT1* (E17K) mutation stimulates migration and invasion of human lung epithelial cells, promoting their oncogenic and metastatic potential [42,51,54].

Nevertheless, *AKT1* alteration is infrequent in PC [41,55,56,57]. For instance, previous studies reported a low frequency of the *AKT1* (E17K) point mutation in PC [40,41,56,57]. On the other hand, some studies did not report *AKT1* (E17K) mutation in PC cases [55]. Consistently, our findings did not report any mutation in the E17 position in the studied cohort. Moreover, in the current study, the *AKT1* gene exhibited a considerable frequency of mutations in exon 4 in the studied cohort.

The relatively high frequency of the *AKT1* mutations, particularly in exon 4, exhibited a possible association between PH domain mutations and the risk of PC; these mutations need further functional studies to reveal their molecular impact in the development and pathogenesis of PC. However, a previous study on bladder cancer detected a mutation in the PH domain, namely (E49K), which was demonstrated as an enhancer activation mutation that transforms activity in NIH3T3 cells, proposing cooperative enhancement of the *AKT1* when it tandemly contains E17K hotspot mutation [39]. Both mutations alter glutamic acid to lysine, which is expected to cause a drastic change in the conformation of the AKT1 due to the conversion from negatively charged amino acid to positively charged residue. Consequently, certain missense mutations that are detected in this study are expected to have a high impact on the conformation and maybe the localization of Akt1. For instance, Ser56Phe, Arg41Trp, Phe35Tyr, and Gln43Lys mutations result in the alteration from polar to non-polar, positively charged to non-polar, non-polar to polar, and polar to positively charged residues, respectively. In addition, these mutations were reported in other types of cancers. On the other hand, the synonymous mutations in exon 3 and exon 4 are expected to have a mild effect on the conformation and function of Akt1, yet a clear conclusion requires functional studies on these mutations. Poor clinical outcomes are associated with the activating mutations in the AKT1/PIK3CA pathway, which underlines the importance of *AKT1* oncogenic impact in PC [58]. Therefore, the AKT1/PI3K pathway has been suggested as a target for the treatment of PC as inhibitors of the PH domain (alkylphospholipid, perifosine). In addition, pan-AKT inhibitors are also suggested (ATP-competitive inhibitor GSK690693 (GlaxoSmithKline)) and (MK2206 (Merck, Inc)) in combination with docetaxel [59,60]. However, drug tolerance, toxicity, and resistance are serious concerns against the approval of those treatments. Therefore, the molecular portrait of the critical oncogenes is crucial for the choice of treatment in precision medicine. The presence of genetic alteration in exon 4 of the *AKT1* gene in PC suggests the significance of genetic screening for precise/effective treatment.

The current results pave the route for future studies to understand the potential influence of these two novel mutations on new therapies of PC, such as hormonal therapies, and for studies that aim to search for predictive biomarkers for the efficiency of such novel therapies. Furthermore, the researchers recommend and highlight the importance of establishing a follow-up system in Northern Jordan, which will be a beneficial system, particularly in studying the relation between the presence of the mutations and the risk for relapse of the disease, recurrence, or metastasis.

## 5. Conclusions

In conclusion, there is a high frequency of *AKT1* mutation in PC patients in Jordan, with two novel missense mutations in the PH domain. E17K hotspot mutation was not detected in any tested samples, which underlines the significant role of mutations in other *AKT1* exons in PC development.

## Figures and Tables

**Table 1:** Primer sequences, conditions of thermal cycler used to produce the PCR product for *AKT1* gene exons 3 and 4, and product size.

	Exon 3			Exon 4		
Forward 5′-3′	GTC AGA GAG CTT AGA GGG AT			GGT CTG ACG GGT AGA GTG TG		
Reverse 5′-3′	AGG GCA CAG GCA CTC ACA GA			ACG CAG TGC TTG TTG CTT CG		
Operation	Temp (°C)	Time (second)	Cycles	Temp (°C)	Time (second)	Cycles
Initial denaturation	95	300	1	95	300	1
Denaturation	95	30	40	95	30	45
Annealing	60	30		60	30	
Elongation	72	40		72	40	
Final elongation	72	5 min	1	72	300	1
Product size (bp)	375			309		

**Table 2:** *AKT1* gene mutations in exons 3 and 4.

Mutation Number	Exon	Position	Mutation Type	Amino Acid Change	Codon Change	Recorded Genotype (N)
rs1180873760	3	c.36G > A	Synonymous Variant	Leu12	CTG > CTA	G/A
rs371534192	3	c.6C > T	Synonymous Variant	Ser2	AGC > AGT	C/T
rs2140948571	4	c.168T > A	Synonymous Variant	Ser56	TCT > TCA	T/A
rs370287382	4	c.120G > A	Synonymous Variant	Glu40	GAG > GAA	G/A
rs2140949629	4	c.80T > A	Missense Variant	Phe27Tyr	TTC > TAC	T/A *
rs749781543	4	c.81C > A	Missense Variant	Phe27Leu	TTC > TTA	C/A *
rs1892948718	4	c.172G > A	Missense Variant	Ala58Thr	GCG > ACG	C/A
rs2140948579	4	c.167C > T	Missense Variant	Ser56Phe	TCT > TTT	C/T
rs753765116	4	c.121C > T	Missense Variant	Arg41Trp	CGG > TGG	T/T
rs2140949369	4	c.103T > A	Missense Variant	Phe35Leu	TTC > ATC	T/A
rs201636005	4	c.96T > A	Missense Variant	Asp32Glu	GAT > GAA	T/A *
rs2140949360	4	c.104T > C	Missense Variant	Phe35Tyr	TTC > TAC	T/C *
COSV62573749 COSM6196752	4	c.127C > A	Missense Variant	Gln43Lys	CAG > AAG	C/A

* means missense mutation.

**Table 3:** The positions and amino acid changes of novel mutations in exon 4.

Exon	Position	Amino Acid Change	Codon Change	Recorded Genotype	Poly-Phen2 Prediction *
4	c.157A > T	Asn53Tyr	TCA > TCT	A/T	Probably damaging
4	c.175C > A	Gln59Lys	CAG > AAG	C/A	Benign

* Using the PolyPhen2 website (http://genetics.bwh.harvard.edu/pph2/ accessed on 1 April 2024).

**Table 4:** Correlation between PSA with age and Gleason score/exon 4 mutations.

Variables							
	Prostate-Specific Antigen (PSA) ng/mL						*p* Value
	<4	4–10	10–20	20–100	>100	No Data	
Age group							0.15
<70 years	4	5	2	6	3	11	
70–80 years	4	5	2	9	2	16	
>80 years	0	2	3	2	2	6	
Gleason score							0.24
<7	1	4	1	2	0	7	
=7	3	4	2	3	1	12	
>7	4	2	4	11	6	14	
No data	1	1	0	0	0	1	

**Table 5:** The comparison between wild-type samples (W) and mutant samples (M) based on clinical and pathological characteristics (age, PSA level, and Gleason score); Chi-square.

Parameter	Category	*AKT1* Exon 4 Mutation Status			*p* Value
		N	M	W	
Age years	<70	30	7	23	0.9645
	70–80	39	9	30	
	>80	15	3	12	
PSA level ng/mL	<4	8	1	7	0.7306
	4–20	19	5	14	
	>20	23	5	18	
Gleason score	<7	15	2	13	0.1313
	=7	25	9	16	
	>7	41	7	34	

## Data Availability

The data used to generate the results presented in the paper are available and can be shared upon reasonable request to the corresponding authors.
